# Mass Spectrometry and Multiplex Antigen Assays to Assess Microbial Quality and Toxin Production of *Staphylococcus aureus* Strains Isolated from Clinical and Food Samples

**DOI:** 10.1155/2014/485620

**Published:** 2014-05-29

**Authors:** Paul Attien, Haziz Sina, Wardi Moussaoui, Kiran Zimmermann, Thomas Dadié, Daniel Keller, Philippe Riegel, Vincent Edoh, Simeon O. Kotchoni, Marcellin Djè, Gilles Prévost, Lamine Baba-Moussa

**Affiliations:** ^1^Laboratoire de Biotechnologie et Microbiologie des Aliments, Faculté des Sciences et Technologies des Aliments, Université Nangui Abroguoua, BP 801 Abidjan 02, Cote D'Ivoire; ^2^Laboratoire de Biologie et de Typage Moléculaire en Microbiologie, Faculté des Sciences et Techniques, Université d'Abomey-Calavi, 05 BP 1604 Cotonou, Benin; ^3^Laboratoire de Bactériologie et Virologie, Faculté des Sciences Médicales, Centre Hospitalier et Universitaire de Treichville, BP V3 Abidjan, Cote D'Ivoire; ^4^Université de Strasbourg (CHRU Strasbourg), Fédération de Médecine Translationnelle de Strasbourg, EA 7290 Virulence Bactérienne Précoce, Institut de Bactériologie, 3 rue Koeberlé, 67000 Strasbourg, France; ^5^Department of Biology and Center for Computational and Integrative Biology, Rutgers University, 315 Penn Street, Camden, NJ 08102, USA

## Abstract

The aim of our study was to investigate the microbial quality of meat products and on some clinical samples in Abidjan focused on *Staphylococcus* genus and the toxin production profile of *Staphylococcus aureus* (*S. aureus*) isolated. Bacteria were collected from 240 samples of three meat products sold in Abidjan and 180 samples issued from clinical infections. The strains were identified by both microbiological and MALDI-TOF-MS methods. The susceptibility to antibiotics was determined by the disc diffusion method. The production of Panton-Valentine Leukocidin, LukE/D, and epidermolysins was screened using radial gel immunodiffusion. The production of staphylococcal enterotoxins and TSST-1 was screened by a Bio-Plex Assay. We observed that 96/240 of meat samples and 32/180 of clinical samples were contaminated by *Staphylococcus*. Eleven species were isolated from meats and 4 from clinical samples. Forty-two *S. aureus* strains were isolated from ours samples. Variability of resistance was observed for most of the tested antibiotics but none of the strains displays a resistance to imipenem and quinolones. We observed that 89% of clinical *S. aureus* were resistant to methicillin against 58% for those issued from meat products. All *S. aureus* isolates issued from meat products produce epidermolysins whereas none of the clinical strains produced these toxins. The enterotoxins were variably produced by both clinical and meat product samples.

## 1. Introduction

*S. aureus* is a bacterial pathogen distributed worldwide and a leading cause of morbidity and mortality.* S. aureus* is the most abundant member of the indigenous flora of human skin [1]. It causes a variety of infections ranging from mild to severe diseases, life-threatening conditions [2]. This versatile pathogen has evolved to a remarkable ability to resist antibiotics such as methicillin and other beta-lactams, glycopeptides, fluoroquinolones, and aminoglycosides, complicating the management of diseases [3].

Indeed, until the 1990s, methicillin resistance was recognized as a specific trait of healthcare-associated* S. aureus* (HA-MRSA), which was first described in the early 1960s [4]. But, methicillin-resistant* S. aureus* (MRSA) strains spread throughout the world, first in hospital settings, but also in the community [5]. It has been reported that 8% of healthy human adults are colonized with MRSA [6]. Apart from resistance, the pathogenicity of* S. aureus* is related to exhaustive vast arsenal of virulence factors and toxins that mainly counteract innate immunity to avoid further adaptive immunity [7].

Among those factors are exotoxins responsible for human infections such as exfoliative toxins (ETs), toxic shock syndrome toxin-1 (TSST-1), staphylococcal enterotoxins (SEs), leukocidins (PVL, LukE/D) and haemolysins (*α*, *β*, *γ*, *δ*) [8]. Enterotoxins are often the cause of food poisoning [9] while exfoliative toxins (also call epidermolysins) act upon the skin [10].* S. aureus* can be found as well in clinical sample as on ready-to-eat foods. Thus,* S. aureus* can be isolated in most of the biological samples [11, 12].

Concerning foods, according to Dennaï et al. [13] and Fosse et al. [14], meat has been traditionally regarded as a vehicle for many foodborne diseases in humans. Its hygienic quality depends on the contamination occurring during slaughtering and cutting process and the development and growth of these biocontaminants during cooling, storage, and distribution [13, 15].

In many tropical countries foods are commonly sold at all public places and roadside shops. However, in view of their ready consumption, quick methods of cleaning and handling them might often constitute a public health threat. In addition, the slaughterhouses are one of the main critical points of meat hygiene and they are considered to be the stage where the greatest opportunities of contamination may occur [16]. According to Jouve [17], 80% to 90% of the microflora of meat reaching the consumers resulted from contamination occurring at the slaughterhouse. The pathogenicity and the resistance profile of Ivoirians* Staphylococcus* are not yet known because strains are not clearly identified and defined. This lack of information makes the establishment of the impact of such bacteria in the pathologies where they are involved difficult. The aim of our present study was to investigate the resistance to antibiotic and toxin production of* S. aureus* isolated from clinical samples and meat products collected in Abidjan, Cote D'Ivoire.

## 2. Material and Methods

### 2.1. Sample Collection

#### 2.1.1. Foods Samples

From November 2009 to March 2011, three kinds of meat (beef, pork, and chicken) were collected in the four most popular sectors of Abidjan (Abobo, Yopougon, Adjamé, and Treichville). At each sector, four places were selected regarding their high diurnal and nocturnal people frequentation. Meat products samples were collected from street sellers as braised meat. One sample of each kind of meat (beef, pork, and chicken) was collected five times (one per month) at each site. For the whole study, 240 samples with 80 samples (20 samples per site) of each kind of meat products were collected. The samples were collected in sterile Stomacher papers then carried to laboratory in icebox at <4°C.

#### 2.1.2. Clinical Samples

The clinical strains were collected from 180 biological samples and carried to the bacteriology unit of the University Hospital of Treichville (Cote D'Ivoire) for various routine bacteriological screenings, from November 2009 to March 2011. According to their site of collection, the collected biological samples were pooled in three groups: (i) patients having pus and serositises, (ii) patient with urogenital infections (urine, vaginal, and urethral), and (iii) mucous membrane (skin and nostril) of healthy persons. During these seventeen months of our study, seventy samples of each group were collected. A part of urine samples we collected in sterile tubes, the other samples were collected with swabs.

### 2.2. Microbiological Analysis

#### 2.2.1. Foods Samples

Once at the laboratory, 10 g of each food sample was homogenized in 90 mL of sterile bacteriological peptone (Oxoid, Hampshire, UK) and then was incubated at 37°C for 1 to 3 h [17]. To perform the isolation of* Staphylococcus* strains, 0.1 mL of serial decimal dilutions was plated in duplicate on Baird-Parker Agar medium (Biolab, South Africa) with 50 mL egg-yolk tellurite emulsion (Merck, Darmstadt, Germany) and incubated at 37°C for 48 h.

#### 2.2.2. Clinical Samples

The swabs were directly streaked on specific medium. For the urine samples, 1 *μ*L was streaked onto surface of blood agar and Cystine-Lactose Electrolyte Deficient (CLED) agar using standard wire loop. All the plates were incubated at 37°C for 24 h.

### 2.3. Microorganism Identification

Standard microbiological methods for microorganism's identification were used [18]. Then,* S. aureus* identification was based on Gram staining, morphology, catalase positivity (ID color Catalase; bioMérieux, Marcy l'Etoile, France), agglutination in the Pastorex Staph Plus test (Bio-Rad, Marnes la Coquette, France), and free coagulase production with lyophilized rabbit plasma [19]. Finally, the isolates were confirmed by API Staph (bioMérieux, Marcy l'Etoile, France).

### 2.4. MALDI-TOF Mass Spectrometry

MALDI-TOF mass spectrometry was used to confirm the microbial identification. The direct identification of bacteria by the MALDI-TOF/MS was serially processed in parallel of the routine protocol. The bacterial pellet was treated with the standard ethanol/formic acid protein extraction protocol before MALDI-TOF identification using a Biflex III mass spectrometer and Flex-analysis, MALDI-Biotyper, software solutions (Biotyper System, Bruker Daltonics) for analysis of acquired data [20, 21].

### 2.5. Antibiotics Susceptibility

Antimicrobial susceptibility was determined by the disc diffusion method of Kirby-Bauer on agar Mueller-Hinton (bioMérieux, Marcy l'Etoile, France) as recommended by the Antibiogram Committee of the French Microbiology Society [22]. After 24 h at 37°C, inhibition zone was measured. For susceptibility to oxacillin, inoculum of 10^7^ CFU/mL was prepared, and the plate was incubated at 37°C for 24 h on Mueller-Hinton agar + 2% NaCl. The tested antibiotics (Bio-Rad, Marne la Coquette, France) were Pristinamycin, Erythromycin, Lincomycin, Oxacillin, Amoxicillin, Ceftriaxone, Gentamicin, Tobramycin, Sisomicin, Oxytétracycline, Tetracycline, Trimethoprim/sulfonamides, Cefotaxime, Ofloxacine, Pefloxacin, Vancomycin, Rifampicin, and Imipenem.

### 2.6. Toxins Production

#### 2.6.1. Phenotypic Detection of Toxins

For the phenotypic detection of toxins radial gel immunodiffusion was performed. The production of Panton-Valentine Leukocidin (PVL) and epidermolysins A (ETA) and B (ETB) was evidenced from culture supernatants after 18 h of growth in Yeast Casamino-acid Pyruvate (YCP) medium [23] by radial gel immunodiffusion in 0.6% (wt/vol) agarose with component-specific rabbit polyclonal and affinity-purified antibodies [24, 25].

#### 2.6.2. Staphylococcal Enterotoxins Production by Bio-Plex Assay (xMAP Multiplex Assay)

The centrifuged supernatant (3 mL) of* S. aureus* grown on BHI at 37°C (night) was recovered and diluted 1/2 in TBS-Tween 20 (0.05%)—nonspecific rabbit IgG at 100 *μ*g/mL, and incubated for 30 min at room temperature (25°C). The Bio-Plex Assays consisted of three incubation steps that were performed into flat-bottom Multiscreen microplates (pores diameter = 1.2 *μ*m, Millipore) according to the previously describe method [26]. Any steps were separated by three washes into TBS-Tween 20. Enterotoxin SEA, SEB, SEC, SED, SEE, SEG, SEH, and SEI were screening by this method in this study.

### 2.7. Data Analysis

For comparison tests of positive isolates in various samples, Student's *t*-test, and Fischer's test were used for lower number series (GraphPad Prism 5). *P* < 0.05 was considered statistically significant.

## 3. Results

### 3.1. Bacterial Identification

Our data reveal the presence of* Staphylococcus* sp. in both clinical and meat product samples. Globally, in our study (Table 1),* S. aureus* is isolated in 42/128 (32.81%) followed by* S. sciuri *(28.90%). Nevertheless, there were isolated 11* Staphylococcus* species in meat products and 5 species in clinical tested samples (Table 1). Regarding the 96 meat samples (over 240 samples) contaminated by* Staphylococcus* species, the isolated rate of the 11 species was* S. sciuri *(33%),* S. aureus *(20%),* S. simulans *(16%),* S. xylosus *(12.50%),* S. cohnii *(5%),* S. lentus *(4%),* S. haemolyticus *(3%),* S. saprophyticus *(2%),* S. capitis *(2%),* S. succinus *(1%), and* S. equorum *(1%). For the clinical samples, among the 32 contaminated ones (over 180 samples), the four following species were isolated:* S. aureus, S. xylosus*,* S. haemolyticus, *and* S. capitis* (Table 1).

### 3.2. Antibiotics Susceptibility

**Figure 1:**
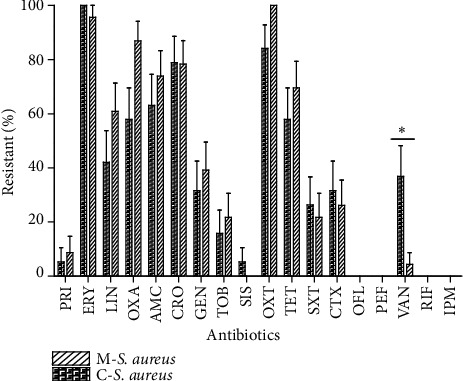
Resistance profile to 18 antibiotics of* Staphylococcus aureus *strains isolated from clinical and meat samples. PRIS: Pristinamycin; ERY: Erythromycin; LIN: Lincomycin; OXA: Oxacillin; AMC: Amoxicillin; CRO: Ceftriaxone; GEN: Gentamicin; TOB: Tobramycin; SIS: sisomicin; OXT: Oxytetracycline; TET: Tetracycline; SXT: Trimethoprim/sulfonamides; CTX: Cefotaxime; OFL: Ofloxacine; PEF: Pefloxacin; VAN: Vancomycin; RIF: Rifampicin; and IPM: Imipenem. ^*^P < 0.05.

The susceptibility of* S. aureus* strains varies depending on antibiotic tested and the origin of the strains (Figure 1). We thus observe for the clinical strains high resistance level to Oxy-tetracycline (100%), Erythromycin (97%), Oxacillin (89%), and Ceftriaxone (81%). There was a very low resistance level of the clinical strain to 7/18 antibiotics: Imipenem (0%), Rifampicin (0%), Ofloxacin (0%), Pefloxacin (0%), Sisomicin (0%), Pristinamycin, and Vancomycin. The same resistance profile was observed with strains isolated from meats but there was variation of resistance proportions. Then, we observed the highest resistance level with Erythromycin (100%), Oxytetracycline (84%), Ceftriaxone (79%), and Oxacillin (58%). We therefor observed a very high level of vancomycin resistance (37%) among meat products isolates. This proportion is statistically higher than the 4% of resistance among clinical strains (*P* < 0.05). The most active antibiotics on* S. aureus* isolated from meat product were Imipenem (0%), Rifampicin (0%), Ofloxacin (0%), Pefloxacin (0%), Pristinamycin, and Sisomicin.

### 3.3. Production of Toxins

**Figure 2:**
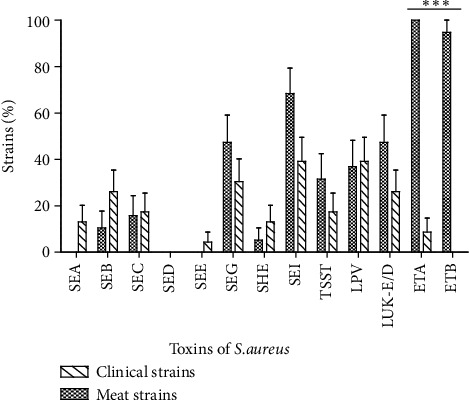
Profile of toxins production by the* S. aureus* strains isolated from clinical and meat samples. SEA: staphylococcal enterotoxin A; SEB: staphylococcal enterotoxin B; SEC: staphylococcal enterotoxin C; SED: staphylococcal enterotoxin D; SEE: staphylococcal enterotoxin E; SEG: staphylococcal enterotoxin G; SEH: staphylococcal enterotoxin H; SEI: staphylococcal enterotoxin I; TSST: Toxic-shock syndrome Toxin; PVL: Panton-Valentine Leukocidin; Luk-E/D: Leukotoxin E/D; ETA: Exfoliative Toxin A; ETB: Exfoliative Toxin B. ^***^P < 0.0001.

Our data display a variability of toxins production according to origin (*P* < 0.0001). Globally,* S. aureus* isolated from meat products produced more toxins than those isolated from clinical samples (Figure 2). The epidermolysins were exclusively produced by meat isolated strains (*P* < 0.0001).

**Figure 3:**
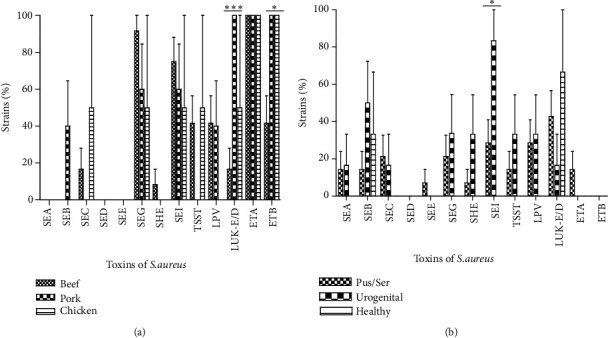
Specificity of the toxins production by the* S. aureus* strains isolated from meat (a) and clinical (b) samples according to their origin. SEA: staphylococcal enterotoxin A; SEB: staphylococcal enterotoxin B; SEC: staphylococcal enterotoxin C; SED: staphylococcal enterotoxin D; SEE: staphylococcal enterotoxin E; SEG: staphylococcal enterotoxin G; SEH: staphylococcal enterotoxin H; SEI: staphylococcal enterotoxin I; TSST: Toxic-shock syndrome Toxin; PVL: Panton-Valentine Leukocidin; Luk-E/D: Leukotoxin E/D, ETA: Exfoliative Toxin A; ETB: Exfoliative Toxin B. ^*^P < 0.05, ^***^P < 0.0001.

Thus, the food isolates produced 10 of the 13 tested toxins. The epidermolysins A (100%) and B (89.5%) were the most produced followed by enterotoxins I (68.4%) G (47.4%). Among the strains isolated from meats, the toxins production was highly different (*P* < 0.0001). Regarding the kind of meat, we observed a difference of toxins production. Indeed, isolates from beef products produced 9 over the 13 sought toxins when those isolated from pork and chicken meat produced 7 over 13 (Figure 3(a)).

Considering the clinical strains, we noted the production of 11 of the 13 tested toxins. For these clinical strains, PVL and enterotoxin I were the most often produced toxin (39.1%). Most of the toxins were produced by few clinical strains (Figure 2). According to the origin of the strains, we observed a slight variation in toxins production. Thus, the PVL was not produced by the isolates issued from healthy persons and the highest level was observed with the samples from pus and serositises (Figure 3(b)).

## 4. Discussion

### 4.1. Bacterial Identification

The first part of our work was to identify the* Staphylococcus* species isolated from clinical samples collected in some hospitals of Abidjan (Cote D'Ivoire) and three kinds of meat sold in Abidjan. Table 1 indicates that food samples were the most contaminated by* S. aureus* strains, despite being cooked at the sampling time. Indeed, from meat samples, 11 different species were isolated whereas in clinical samples we isolated 5 species. Then, 10 different coagulase negative staphylococci (CNS) species were found in our meat samples and 4 in the clinical ones. The number of species identified in our study is higher than those isolated in Croatia in fermented sausage [27]. The nature of meat product can explain the observed difference in terms of number of species. In fact, the process used to prepare fermented sausages needs more steps than our meat sample. Then, during the manufacturing of fermented sausages, some species are destroyed. The great number of coagulase negative staphylococci (CNS) in meat samples (10 species) in comparison with clinical ones [28] can be explained by the fact that those CNS are commonly used for meat fermentation. Indeed, CNS such as* S. xylosus* [29],* S. equorum,* and* S. saprophyticus* [30–32] have been reported all over the world in fermented meats as starter cultures [33, 34]. The CNS species are isolated from both meat chain production and final meat products [35]. Then, we can estimate that most of the CNS isolates are useful in the fermentation process of the meats products sold in the streets of Abidjan. These CNS are potentially less pathogenic and may be part of the skin flora, and they hold rarely toxigenic component [36]; we must nevertheless pay attention because these bacterial develop resistance to many antibiotic molecules [37].

### 4.2. Antibiotics Susceptibility

The second part of our work aims at studying the antibiotics susceptibility of* S. aureus* isolated from clinical and meat samples to 18 antibiotics. Figure 1 showing the resistance profile of the 42* S. aureus* strains indicates variability according to the antibiotics and the origin of the strains. For both clinical and food isolated* S. aureus* strains, the highest resistance levels were observed with Oxy-tetracycline, Erythromycin, Oxacillin, and Ceftriaxone. Our data display a high resistance level to macrolides and *β*-Lactamines. The observed high proportions can be the effect of excessive use of invalid antibiotics and traditional medicine out of the hospital area; that contributes to selecting resistant strains in the community.

Our data show a high proportion of* S. aureus* strains resistant to oxacillin independently of their origin. This proportion of 89% for clinical strains and 58% for meat strains observed increase steadily in Cote D'Ivoire. Indeed, in 2012 Zinzendorf et al. [38] observed less than 20% of* S. aureus *resistance to methicillin in a Military Hospital at Abidjan (Cote D'Ivoire). The observed gap with this study can be explained by the variability of our stains. Indeed, our strains were isolated from various kinds of clinical and meat samples. Another explanation, that is, the difference of methodology, may be outlined because these authors used the molecular approach, whereas we used the disc diffusion method. Then, analyzing this result, the efficacy of the formerly indicated that molecule against* S. aureus* strains is decreasing [39].

A high level of vancomycin resistance (37%) among meat products isolates in comparison to the clinical isolates (*P* < 0.05) was observed. In hospitals, where higher selective pressure is normally present, vancomycin is one of the antibiotics used to treat multiresistance* S. aureus* strains. This surprising result may be an effect of invalid use of antibiotics to treat suspected food poisoning or farm animal infections in this area. Another possible reason of this difference may be explained by the fact that, in this area, farmers currently use an excess of the glycopeptides avoparcin as a growth promoter in food-producing animals indicating that these animals might be a potential reservoir for vancomycin resistance determinants [40–42].

### 4.3. Production of Toxins

Figure 2 indicated the distribution of 13 toxins usually produced by* S. aureus* strains according to their origin (*P* < 0.0001). Indeed, comparing with their origins (clinical and meat), clinical isolated strains produce 11 of the 13 sought toxins when meat isolated ones produced 10 of 13. Concerning the isolates issued from meat products, the epidermolysins A (100%) and B (89.5%) were the most produced followed by enterotoxins I (68.4%) and G (47.4%). Among the strains isolated from meats, the toxins production was highly different (*P* < 0.0001). The high level of epidermolysins indicates that these isolates in meat may be originated from an upstream phase of preparation, such as the handling of the meat sealers or the unsterile containers. We should remark that the meats are usually sold in papers package (mainly cement package paper). Indeed, epidermolysins are known to be serine active proteases with their activity highly specialized on desmoglein-1, an important protein of child epidermis [43, 44]. Regarding the kind of meat, we observed that strains isolated from beefs produced 2 toxins (SEA and SEE) that were not produced by those isolated from pork and chicken meat (Figure 3(a)). Then, the high level of* S. aureus* producing epidermolysins in the beef samples can be explained by slaughtering contamination. In fact, beef consumed parts are generally separate from the animal skin which constitutes a filter of microorganisms. After removing the skin of beef, the consumed parts become exposed to the sellers' manual contamination during the transformation process. On the back of the epidermolysins production, we observe that enterotoxins and TSST-1 are strongly produced. These observations must attract our attention from probable food poisoning further to the consumption of meat sold in Abidjan streets. Indeed, enterotoxins are known to be associated with food poisoning [45–47]; they are also known to have superantigenic and emetic activities. Clinically, staphylococcal infection is a frequent cause of foodborne gastroenteritis in the world [48], following the ingestion of staphylococcal enterotoxins [49].

For the clinical strains, PVL and enterotoxin I were the most produced toxins (39.1%). Most of the toxins were produced by few clinical isolates (Figure 2). Considering the origin of the isolates, we observe a slight variation on toxins production. Thus, the PVL and enterotoxin I was not produced by the strains isolated from healthy persons, but the highest incidence was observed with the samples of pus and serosities (Figure 3(b)). PVL appears to be a primordial toxin of clinical* S. aureus* isolates and particularly from skin, soft tissues, and bone-related infections [50]. Making a comparison with strains isolated from meats, those isolated from clinical field appear more pathogenic. It was documented that positive PVL* S. aureus* strains are more pathogenic than negative PVL ones [51, 52]. In fact, the cell lysis spectrum of PVL affects directly the monocytes, macrophages, polynuclear neutrophils, and the metamyelocytes and some, at least, neurons [53, 54], although the erythrocytes are not lysed in their presence [55].

## 5. Conclusion

Human infections and meat products accommodate many* Staphylococcus* lineages. The susceptibility to antibiotics indicated the highest level of methicillin resistance among the* S. aureus* isolated from both clinical samples. The toxins production by* S. aureus* reveals that Panton-Valentine Leukotoxin may be the most frequently produced toxin by clinical strains, whereas meat products were most often contaminated by epidermolysins and enterotoxins producers. Through the ability of strains isolated from meat products to produce enterotoxins, we, thus, demonstrate that meats sold in the streets of Abidjan can potentially be a source of food poisoning. This study should be deepened by studying in this area the direct relation between the street food* S. aureus *isolates and clinical infections suck like diarrhea using the genotyping.

## Figures and Tables

**Table 1:** Contamination level of meat products and clinical samples collected in Abidjan, Cote D'Ivoire.

Species	Percentage of meat samples contamination (*n* = 96)			Percentage of clinical samples contamination (*n* = 32)			Total (*n* = 128)
	Beef (*n* = 27)	Pork (*n* = 22)	Chickens (*n* = 47)	Pus/Serositises (*n* = 19)	Urogenital (*n* = 07)	Healthy persons (*n* = 06)	
*S. sciuri *	9% (9)	4% (4)	20% (19)	6% (2)	0% (0)	9% (3)	**28.9% (37)**
*S. aureus *	13% (12)	5% (5)	2% (2)	44% (14)	19% (6)	9% (3)	**32.8% (42)**
*S. simulans *	1% (1)	6% (6)	8% (8)	0% (0)	0% (0)	0% (0)	**11.7% (15)**
*S. xylosus *	1% (1)	4% (4)	7% (7)	0% (0)	3% (1)	0% (0)	**10% (13)**
*S. cohnii *	0% (0)	1% (1)	4% (4)	0% (0)	0% (0)	0% (0)	**4% (5)**
*S. lentus *	0% (0)	1% (1)	3% (3)	0% (0)	0% (0)	0% (0)	**3% (4)**
*S. haemolyticus *	3% (3)	0% (0)	0% (0)	6% (2)	0% (0)	0% (0)	**4% (5)**
*S. saprophyticus *	1% (1)	1% (1)	0% (0)	0% (0)	0% (0)	0% (0)	**1.6% (2)**
*S. capitis *	0% (0)	0% (0)	2% (2)	3% (1)	0% (0)	0% (0)	**2% (3)**
*S. succinus *	0% (0)	0% (0)	1% (1)	0% (0)	0% (0)	0% (0)	**1% (1)**
*S. equorum *	0% (0)	0% (0)	1% (1)	0% (0)	0% (0)	0% (0)	**1% (1)**
